# Downregulation of the phosphatase PHLPP1 contributes to NNK-induced malignant transformation of human bronchial epithelial cells (HBECs)

**DOI:** 10.1016/j.jbc.2025.108221

**Published:** 2025-01-23

**Authors:** Xuelei Liu, Shirui Huang, Xiaozhen Gu, Ziyi Yang, Jiajun Xiu, Xiaodan Xu, Yaxin Cao, Jingjing Wang, Yunping Zhao, Minggang Peng, Zhongxian Tian, Xiaohui Hua, Hui-Li Wang, Chuanshu Huang

**Affiliations:** 1 Key Laboratory of Medicine, Ministry of Education, School of Laboratory Medicine and Life Sciences, Wenzhou Medical University, Wenzhou, Zhejiang, China; 2 Oujiang Laboratory (Zhejiang Lab for Regenerative Medicine, Vision and Brain Health), Wenzhou, Zhejiang, China; 3 Engineering Research Center of Bio-process, Ministry of Education, Hefei University of Technology, Hefei, Anhui, PR China; 4 School of Food and Biological Engineering, Hefei University of Technology, Hefei, Anhui, PR China; 5 Department of Obstetrics and Gynecology, Union Hospital, Tongji Medical College, Huazhong University of Science and Technology, Wuhan, Hubei, China

**Keywords:** NNK, PHLPP1, PTEN, PP2AC, miR-613, lung carcinogenesis

## Abstract

Cigarette smoking (CS) is one of the greatest health concerns, which can cause lung cancer. 4-(Methylnitrosamino)-1-(3-pyridyl)-1-butanone (NNK), a tobacco-specific nitrosamine, has been well-documented for its carcinogenic activity in both epidemiological and laboratory studies. PH domain leucine-rich repeat protein phosphatase 1 (PHLPP1) and phosphatase and tensin homolog (PTEN) are two well-known phosphatase tumor suppressors that have been reported to be downregulated in human lung cancer tissues. However, the effect of NNK exposure on the expression of PHLPP1 and PTEN is unknown, and such effects may be early events leading to lung carcinogenesis. We explored this question in current studies and found that exposure of human bronchial epithelial BEP2D cells to NNK resulted in cell malignant transformation accompanied by a remarkable downregulation of PHLPP1 and PTEN. Such downregulation of PHLPP1 and PTEN was also consistently observed *in vivo* in Cigarette Smoking-exposed mouse lung tissues. Our studies further showed that overexpression of PHLPP1 or PTEN alleviated NNK-induced BEP2D cell transformation. Ectopic expression of PHLPP1 promoted PTEN protein expression, while PTEN overexpression did not affect PHLPP1 expression. Mechanistic studies showed that NNK was able to inhibit PP2A-C activity, which directly attenuated c-Jun phosphorylation at Ser63/73, and subsequently inhibited the PHLPP1 transcription and expression. Further, the downregulation of PHLPP1 led to a reduction of *pten* mRNA stability and expression through the HUR/Jun D/miR-613/NCL axis. Taken together, our studies advance the field of tobacco-induced lung cancer research by uncovering new mechanistic insights and identifying a novel molecular axis that could inform future therapeutic strategies. It also adds a new dimension by exploring the interaction between PHLPP1 and PTEN in the context of tobacco carcinogen exposure.

Cigarettes contain 1000 of chemicals, including over 60 known carcinogens. The Centers for Disease Control and Prevention (CDC) reported that smoking is associated with about 80 to 90 percent of lung cancer deaths in the United States. Cigarette smoking can induce lung adenocarcinomas (1). During the roasting and burning of tobacco, nicotine is converted to nitrosamines through nitrosation (2). Nicotine-derived nitrosamines (NNK) and nitrososonic nicotine (NNN) are recognized carcinogens in humans and animals. NNK has recently been shown to have a significant association with lung cancer, but the mechanism by which NNK induces lung cancer is not fully understood (3). Therefore, current studies explore the molecular mechanisms underlying the lung carcinogenic effects of NNK both *in vitro* and *in vivo*.

Phosphatase and tensin homolog (PTEN) is a phosphatase that can metabolize PIP_3_, the lipid product of PI 3-Kinase, directly opposing the activation of the oncogenic PI3K/AKT/mTOR signaling network (4). PH domain leucine-rich repeat protein phosphatase (PHLPP) is a family of enzymes made up of two isoforms (PHLPP1 and PHLPP2), whose actions modulate intracellular activity *via* the dephosphorylation of specific serine/threonine (Ser/Thr) residues on proteins such as Akt (5). PTEN and PHLPP1 are tumor suppressor genes (6, 7, 8). The reduction of PTEN protein expression increases cancer susceptibility. Previous studies have reported that high levels of estrogen receptor (ER) accumulation in patients with non-small cell lung cancer (NSCLC) positively feedback to activate AKT, which ultimately suppresses the expression of PTEN, leading to cisplatin resistance in NSCLC (9). PTEN deletion increases susceptibility to lung cancer induced by tobacco carcinogen NNK, suggesting an important role of PTEN in tobacco-initiated lung cancer development (10). Additionally, it was reported that PHLPP1 acts as a tumor suppressor in cancer cells (11). However, nothing is known about the molecular mechanisms underlying PTEN and PHLPP1 in NNK-induced lung carcinogenesis and whether there is a crosstalk between PHLPP1 and PTEN in the processes of chronic NNK-lung carcinogenesis. Therefore, we address these questions in the current studies.

Akt is a serine/threonine kinase that is activated by phosphorylation at Thr308 or Ser473 (12). Conversely, PTEN phosphatase plays a major negative regulatory role in the PI3K/Akt signal pathway (13). The PTEN/Akt pathway plays a vital role in regulating cell apoptosis, metabolism, and cell growth (14, 15, 16, 17). PHLPP1 can also directly target Akt and dephosphorylate the Ser473 site, thereby reducing its activation (18). However, the crosstalk between PTEN and PHLPP1 involved in NNK lung carcinogenesis has not been reported to the best of our knowledge. Here, we addressed this question. Our results showed that NNK exposure led to the downregulation of both PHLPP1 and PTEN protein levels in human bronchial epithelial BEP2D cells and that overexpression of either PTEN or PHLPP1 attenuated NNK-induced transformation of BEP2D cells. Furthermore, we discovered that NNK was able to inhibit PP2A-C activity, which impaired c-Jun phosphorylation at Ser63/73 and subsequently inhibited the PHLPP1 transcription and expression. Additionally, PHLPP1 downregulation resulted in a reduction of *pten* mRNA stability and expression *via* the HUR/Jun D/miR-613/NCL axis.

## Results

### Downregulation of PTEN and PHLPP1 expression was observed as a result of NNK exposure in lung epithelial cells both *in vitro* and *in vivo*

**Figure 1:**
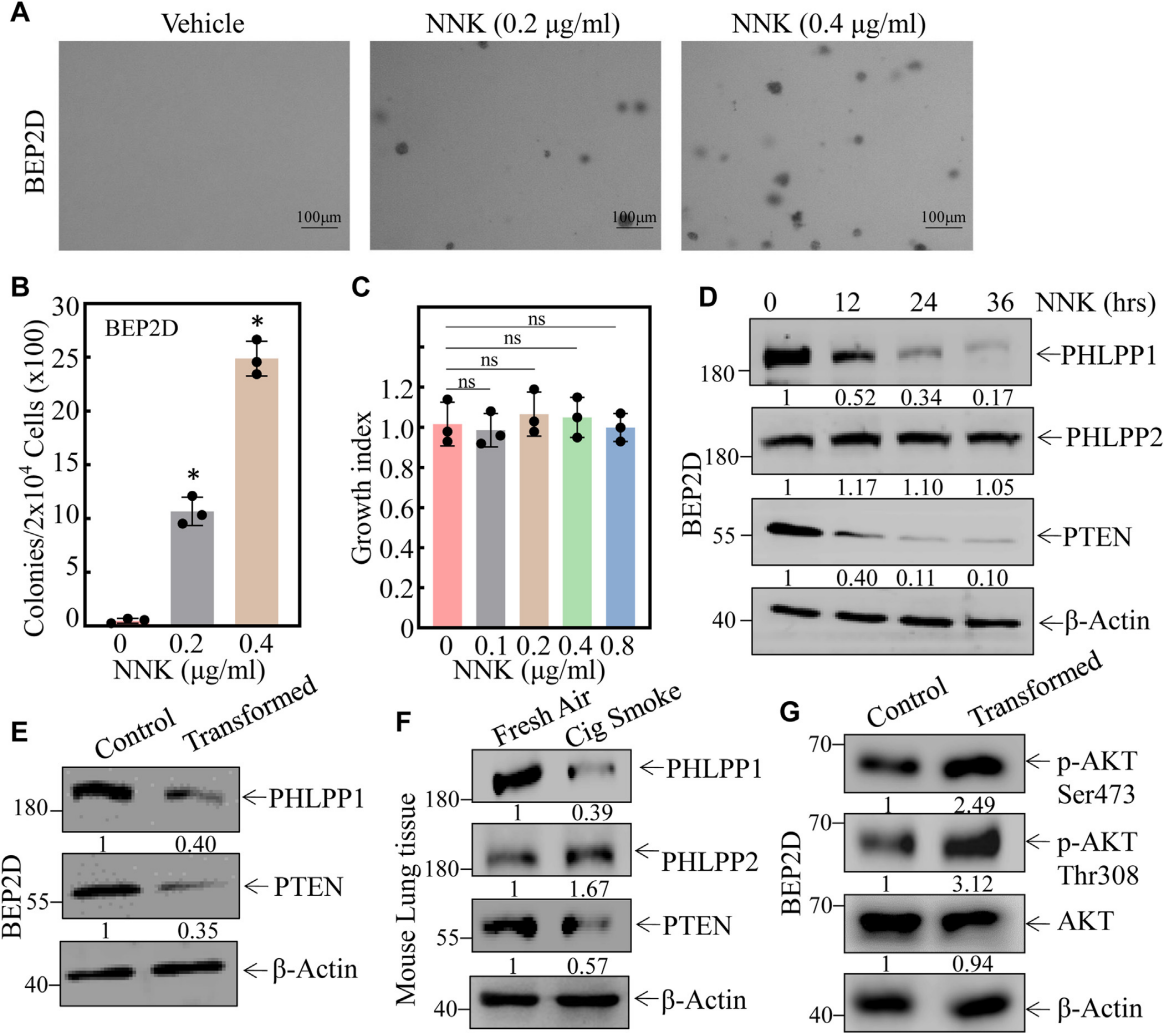
**Downregulation of PTEN and PHLPP1 expression was observed following NNK exposure *in vivo* and *in vitro*.***A* and *B*, chronic exposure to NNK caused BEP2D (Nonsense) cells to acquire anchorage-independent growth in soft agar, a hallmark of malignant cellular transformation. The number of colonies was counted and presented as colonies per 10^4^ seeded cells. The bars shown are mean ± SD from three independent experiments. ∗Significant difference relative to vehicle control (*p* < 0.05). *C*, the cell growth index of BEP2D cells was evaluated after NNK exposure through the CCK-8 assay. ns *p* > 0.05. *D*, Western Blot was employed to determine the expression of phosphatases, including PHLPP1, PHLPP2, and PTEN. β-actin was measured as an internal control. *E*, the relative levels of PHLPP1, PHLPP2, and PTEN were determined in BEP2D(Control) and BEP2D(transformed) cells. β-actin was measured as an internal control. *F*, Western Blot assay was performed to evaluate the expression of PHLPP1, PHLPP2, and PTEN in fresh air-exposed mouse lung tissues and cigarette smoking-exposed mouse lung tissues (FA, fresh air; CS, cigarette smoking; n = 5). β-actin was measured as an internal control. *G*, Western Blot was used to detect the expression of AKT phosphorylation of Ser473 and Thr308 sites in NNK-induced transformed BEP2D cells. β-actin was measured as an internal control. All experiments were performed at least three replicates.

Nicotine-derived nitrosamine ketone (NNK) is a well-documented human lung carcinogen. To investigate the molecular mechanisms underlying NNK-induced lung carcinogenesis, we evaluated the effects of NNK on the malignant transformation of human bronchial epithelial cells and assessed its impact on cell growth. Chronic exposure to NNK induced BEP2D cells to acquire anchorage-independent growth capability in soft agar, a signature of malignant cellular transformation (Fig. 1, *A* and *B*). NNK exposure for 48 h does not affect the cell growth of BEP2D cells (Fig. 1*C*). Such malignant cellular transformation by NNK was accompanied by specific downregulation of PTEN and PHLPP1 in a time-dependent manner, while there was no significant effect on PHLPP2 expression (Fig. 1*D*). Consistently, the downregulation of PHLPP1 and PTEN expression were also observed in chronic NNK-transformed BEP2D cells *in vitro* (Fig. 1*E*) and cigarette smoking-exposed mouse lung tissues *in vivo* (Fig. 1*F*). Given that PTEN and PHLPP1 are both tumor suppressors involved in attenuation of Akt phosphorylation/activation, we next determined whether Akt was implicated in the NNK-induced malignant transformation of human bronchial epithelial cells. The results showed that phosphorylation of Ser473 and Thr308 sites on Akt was upregulated in NNK-transformed BEP2D cells (Fig. 1*G*), indicating that downregulation of PTEN and/or PHLPP1 protein might be associated with Akt activation during NNK-induced lung carcinogenesis.

### Both PTEN and PHLPP1 played a crucial role in the NNK-induced malignant transformation of BEP2D cells

**Figure 2:**
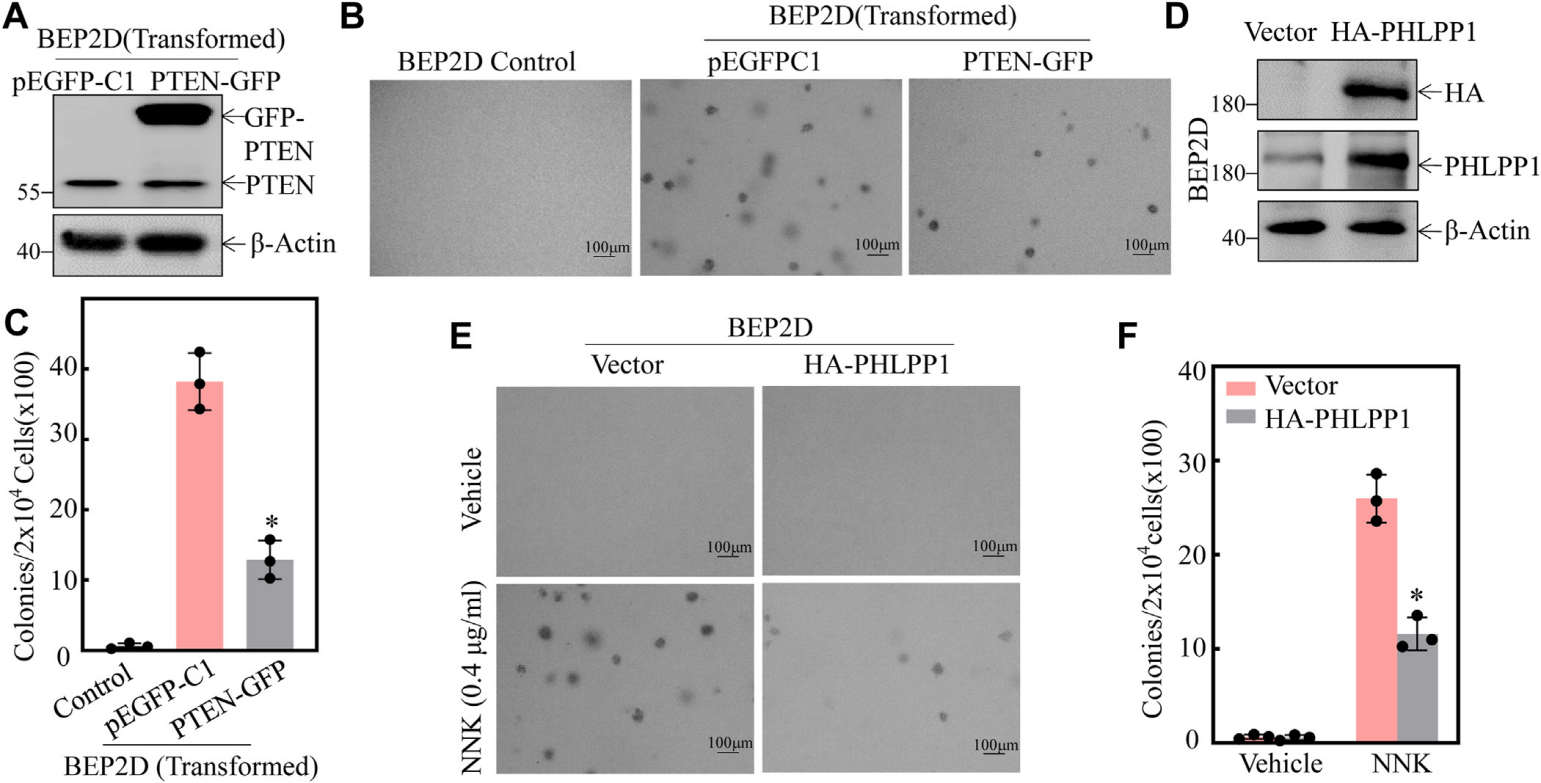
**PTEN and PHLPP1 played a vital role in the NNK-induced malignant transformation of BEP2D cells.***A*, overexpress construct of PTEN was stably transfected into BEP2D(transformed) cells. Western Blot was used to detect the knockdown efficiency of PTEN protein. *B* and *C*, the indicated cells were subjected to soft agar assay. (*B*) The number of colonies was scored and presented as colonies per 10^4^ seeded cells (*C*). The asterisk (∗) indicates a significant decrease in comparison to BEP2D(PEGFPC1) cells (*p* < 0.05). *D*, overexpress construct of the PHLPP1 was stably transfected into BEP2D cells. Western Blot was used to determine HA-PHLPP1 protein expression level. *E* and *F*, the BEP2D(Vector) and BEP2D(HA-PHLPP1) cells were exposed to 400 μg/ml NNK and then subjected to soft agar assay. *E*, the number of colonies was scored and presented as colonies per 10^4^ seeded cells. *F*, the asterisk (∗) indicates a significant change (*p* < 0.05). All experiments were performed at least three replicates.

The previous results suggested that the downregulation of PTEN and/or PHLPP1 protein might be associated with Akt activation during NNK-induced lung carcinogenesis. To test this hypothesis, we stably transfected PTEN-GFP overexpression and HA-PHLPP1-overexpression constructs into BEP2D cells. The stable ectopic expression of the PTEN-GFP transfectant was confirmed as shown in Figure 2*A*. Overexpression of PTEN-GFP alleviated anchorage-independent growth of NNK-transformed BEP2D cells compared to that observed in BEP2D(Nonsense) cells under the same experimental conditions (Fig. 2, *B* and *C*). Moreover, the stable ectopic expression of the HA-PHLPP1 transfectant was confirmed as shown in Figure 2*D*. Stable ectopic expression of HA-PHLPP1 also attenuated BEP2D cell transformation by NNK exposure (Fig. 2, *E* and *F*). These results demonstrated an inhibitory effect of PTEN and PHLPP1 on either NNK-transformed BEP2D cells or NNK-induced transformation of BEP2D cells. Taken together, our results strongly suggested that the downregulation of PTEN and PHLPP1 contributes to human bronchial epithelial cell malignant transformation due to NNK exposure.

### PHLPP1-mediated PTEN protein mRNA stabilization *via* upregulating NCL in human bronchial epithelial cells

**Figure 3:**
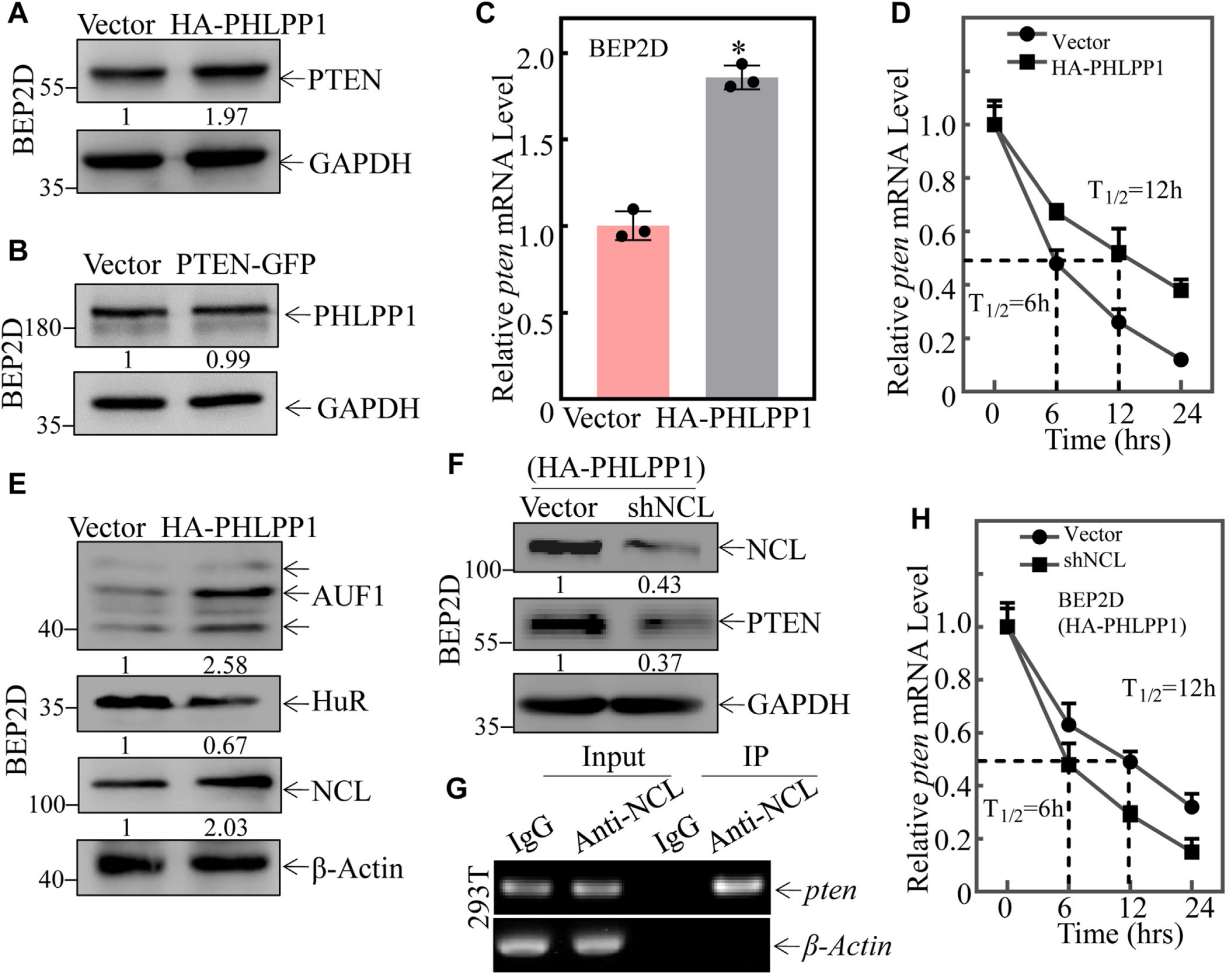
**PHLPP1 upregulates pten mRNA stability through upregulating NCL in BEP2D(HA-PHLPP1) cells compared to BEP2D(Vector) cells.***A*, cell extracts obtained from the BEP2D(Vector) and BEP2D(HA-PHLPP1) transfectants were subjected to Western blot for determination of the PTEN protein expression. β-Actin was used as protein loading control. *B*, cell extracts obtained from the BEP2D(Vector) and BEP2D(PTEN-EGFPC1) transfectants were subjected to Western blot to determination of the PHLPP1 protein expression. β-Actin was used as the protein loading control. *C*, relative *pten* mRNA levels in cells were evaluated using real-time PCR in BEP2D(Vector) and BEP2D(PTEN-EGFPC1) stably transfectants. Bars represent means ± SD from three independent experiments. The asterisk (∗) indicates a significant change (*p* < 0.05). *β-actin* was used as the mRNA loading control. *D*, relative *pten* mRNA stability was evaluated by real-time PCR in BEP2D(Vector) vs. BEP2D(HA-PHLPP1) cells after treatment with Act D for indicated times. *β-actin* was used as the mRNA loading control. *E*, the indicated cell extracts were subjected to Western Blot to determination of AUF1, NCL, and HUR protein expression. β-Actin was used as the protein loading control. *F*, sh-NCL or its control vector was transfected into BEP2D(HA-PHLPP1) cells, and the indicated stable transfectants were subjected to Western blot to determination of NCL and PTEN protein expression as indicated. GAPDH was used as protein loading control. *G*, RNA immunoprecipitation assay using an anti-NCL antibody to test the interaction of NCL with *pten* mRNA in 293T cells. *H*, relative *pten* mRNA stability was evaluated by real-time PCR in the indicated cell cells after treatment with Act D for indicated times. *β-actin* was used as the mRNA loading control. All experiments were performed at least three replicates.

Although exposure to NNK can suppress the expression of PHLPP1 and PTEN, the relationship between PHLPP1 and PTEN has not been explored. To explore their interaction, HA-PHLPP1 or PTEN-GFP ectopic expression transfectants were employed in comparison to their vector transfectants. Overexpression of HA-PHLPP1 in BEP2D cells remarkably upregulated the protein level of PTEN (Fig. 3*A*), while overexpression of PTEN-GFP did not affect the expression of PHLPP1 (Fig. 3*B*), indicating that PTEN serves as a downstream target of PHLPP1.

To further investigate the molecular mechanisms involved in PHLPP1 promoting PTEN expression, *pten* mRNA levels were assessed in BEP2D(Vector) and BEP2D(HA-PHLPP1) transfectants by using Real-time PCR assay. As shown in Figure 3*C*, *pten* mRNA levels were significantly increased in BEP2D(HA-PHLPP1) cells compared to those in BEP2D(Vector) transfectant. To elucidate the mechanism of the increased *pten* mRNA regulated by PHLPP1 overexpression, the stability of *pten* mRNA was evaluated and compared between the two transfectants. The results indicated that overexpression of HA-PHLPP1 significantly increased the stability of *pten* mRNA compared to that observed in BEP2D(Vector) transfectant (Fig. 3*D*). These results reveal that PHLPP1 mediates PTEN protein abundance mainly by enhancing the stabilization of *pten* mRNA.

Several RNA-binding proteins, such as Nucleolin (NCL), Human antigen R protein (HUR), and AU-rich binding factor 1 (AUF1), have been reported to bind their targeted mRNA and modulate mRNA stability (19). We therefore determined whether those RNA-binding proteins were involved in the PHLPP1-regulated stability of *pten* mRNA. The results showed that overexpression of PHLPP1 decreased HUR expression, while it increased the levels of NCL and AUF1 (Fig. 3*E*). HUR and NCL have been reported to stabilize mRNA binding, while AUF1 destabilizes its targeted mRNA when bound to an ARE-containing mRNA(20, 21, 22). Therefore, AUF1 and HUR were excluded as downstream effectors stabilizing *pten* mRNA in PHLPP1. We, therefore, tested the potential role of NCL in PHLPP1 regulating *pten* mRNA stability. The shRNA-specific targeting NCL (shNCL) plasmid was stably transfected into BEP2D(HA-PLPP1) cells to knock down NCL level as identified in Figure 3*F*. Knockdown of NCL also attenuated PTEN protein expression in BEP2D(HA-PHLPP1/shNCL) cells as compared to those observed in BEP2D(HA-PHLPP1) cells (Fig. 3*F*).

To test NCL interaction with *pten* mRNA, an RNA immunoprecipitation assay was performed as shown Figure 3*G*. The results indicated that *pten* mRNA was presented in the immunoprecipitated complex pulled down by anti-NCL antibodies in the intact 293T cells (Fig. 3*G*), demonstrating that NCL does bind with *pten* mRNA. Moreover, to validate the role of NCL in stabilizing *pten* mRNA, HA-PHLPP1(vector) and HA-PHLPP1(shNCL) cells were employed to test the decay rates of *pten* mRNA in the presence of the *de novo* mRNA synthesis inhibitor actinomycin D (Act D). As shown in Figure 3*H*, the knockdown of NCL in HA-PHLPP1 cells dramatically reduced *pten* mRNA stability. These results reveal that PHLPP1 downregulation attenuates *pten* mRNA stability through decreasing NCL expression in NNK—NNK-transformed BEP2D cells.

### NCL protein upregulation was mediated by PHLPP1 attenuating miR-613 expression, which resulted in less miR-613 binding to the 3′-UTR of *ncl* mRNA and increasing in NCL protein translation in human bronchial epithelial cells

**Figure 4:**
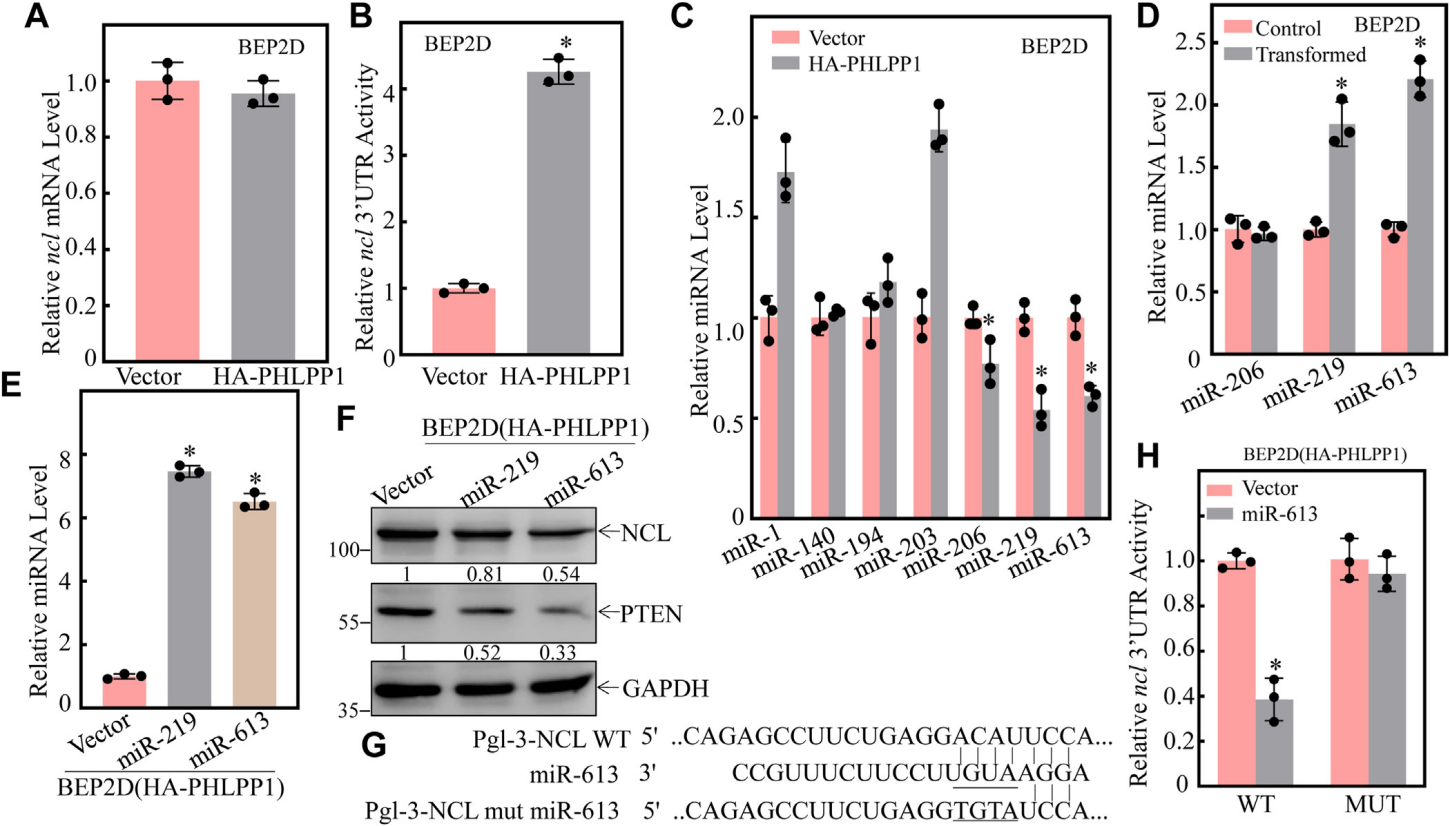
**miR-613 binds to the 3′-UTR of *ncl* mRNA and downregulates its protein expression in the NNK-induced transformaed BEP2D cells.***A*, relative NCL mRNA level was evaluated by real-time PCR in BEP2D(Vector) vs. BEP2D(HA-PHLPP1) cells. *B*, pMIR-NCL 3′-UTR reporters were transiently transfected into the BEP2D(Vector) or BEP2D(HA-PHLPP1) cells, and luciferase activity was evaluated. The asterisk (∗) indicates a significant change (*p* < 0.05). *C*, qPCR was performed to determine the expression of miRNAs in BEP2D (Vector) *vs.* BEP2D(HA-PHLPP1) cells. The asterisk (∗) indicates a significant change (*p* < 0.05). The *U6* was used as the loading control. *D*, qPCR was performed to determine the effect of miRNA expression in BEP2D(Control) *vs.* BEP2D(transformed) cells. The *U6* was used as the loading control. *E*, miR-219 and miR-613 overexpression was identified in BEP2D cells. The asterisk (∗) indicates a significant increase in BEP2D(miR-219) cells *vs*. BEP2D(Vector) or BEP2D(miR-613) cells *vs.* BEP2D(Vector) cells (*p* < 0.05). The *U6* was used as the loading control. *F*, NCL and PTEN expressions were analyzed by Western blot in BEP2D(Vector) vs. BEP2D(miR-219) and BEP2D (miR-613) cells. GAPDH was used as the protein loading control. *G*, schematic of the miR-613 binding site in *ncl* mRNA 3′-UTR regions, and its mutants aligned with miR-613. *H*, the indicated cells were co-transfected with wild-type, mutant NCL 3′-UTR luciferase reporters, and pRL-TK, respectively. Luciferase activity of each transfectant was evaluated and results were presented as relative *ncl* 3′-UTR activity. All experiments were performed at least three replicates.

NCL could be regulated at multiple levels, including transcription, mRNA stability, translation, and protein degradation. To elucidate the molecular mechanisms of NCL upregulation by PHLPP1, we first evaluated *ncl* mRNA levels in the BEP2D(HA-PHLPP1) and BEP2D(vector) cells. The results indicated that the ectopic expression of PHLPP1 didn't influence the *ncl* mRNA levels, excluding the possible regulation of *ncl* mRNA transcription or mRNA stability by PHLPP1 (Fig. 4*A*). It has been well recognized that the mRNA 3′-untranslated region (3′-UTR) could play a regulatory function in protein expression. To explore whether NCL was regulated by PHLPP1 at 3′-UTR level, we transfected the full-length constructs of *ncl* mRNA 3′-UTR luciferase reporter into BEP2D(HA-PHLPP1) and BEP2D(Vector) cells. As shown in Figure 4*B*, HA-PHLPP1 overexpression resulted in a significant upregulation of NCL 3′-UTR activity in comparison to that in BEP2D(Vector) cells. Due to miRNAs regulating the mRNA stability through targeting the mRNA 3′-UTR, we used the miRanda database to analyze potential miRNA-binding sites in *ncl* mRNA 3′-UTR. Seven potential miRNAs, including miR-1, miR-140, miR-194, miR-203, miR-206, miR-219, and miR-613 were predicted. We subsequently evaluated the expression levels of these potential miRNAs by qPCR assay in BEP2D(HA-PHLPP1) and BEP2D(Vector) cells. As shown in Figure 4*C*, PHLPP1 overexpression downregulated miR-206, miR-219, and miR-613 expression, while PHLPP1 upregulated miR-1 and miR-203 levels and had no effect on miR-140 and miR-194 expression. These results reveal that miR-206, miR-219, and miR-613 might have the potential to participate in the regulation of *ncl* mRNA 3′-UTR activity. Thus, we further evaluated the expression of miR-206, miR-219, and miR-613 expression in NNK- transformed BEP2D cells. As shown in Figure 4*D*, the expression of miR-613 and miR-219 in NNK-transformed cells was markedly upregulated in comparison to that observed in untransformed cells, while there was no significant difference of miR-206 between the two transfectants (Fig. 4*D*). So miR-219 and miR-613 expression constructs were stably transfected into BEP2D(HA-PHLPP1) cells and their ectopic expressions were identified as shown in Figure 4*E*. The overexpression of miR-613 remarkedly attenuated NCL protein expression in BEP2D(HA-PHLPP1) cells, whereas miR-219 overexpression had no effect on NCL protein expression (Fig. 4*F*). Moreover, the miR-613 targeted site mutated *ncl* mRNA 3′-UTR luciferase reporter was constructed as shown in Figure 4*G*, and both wild-type (WT) and mutant (MUT) of *ncl* mRNA 3′-UTR luciferase reporters were then transfected into BEP2D(HA-PHLPP1) and BEP2D(Vector) cells, respectively. The results showed that ectopic expression of miR-613 significantly blocked *ncl* mRNA 3′-UTR luciferase reporter activity in WT *ncl* mRNA 3′-UTR luciferase reporter transfectant, whereas miR-613 overexpression lost its such inhibition in the miR-613-binding site mutant (MUT) 3′-UTR luciferase reporter transfectant (Fig. 4*H*), strongly indicating that miR-613 can directly bind to *ncl* mRNA 3′-UTR and repress its mRNA level.

### PHLPP1 overexpression inhibited miR-613 transcription through attenuating *jun d* mRNA stability that was mediated by HUR downregulation

**Figure 5:**
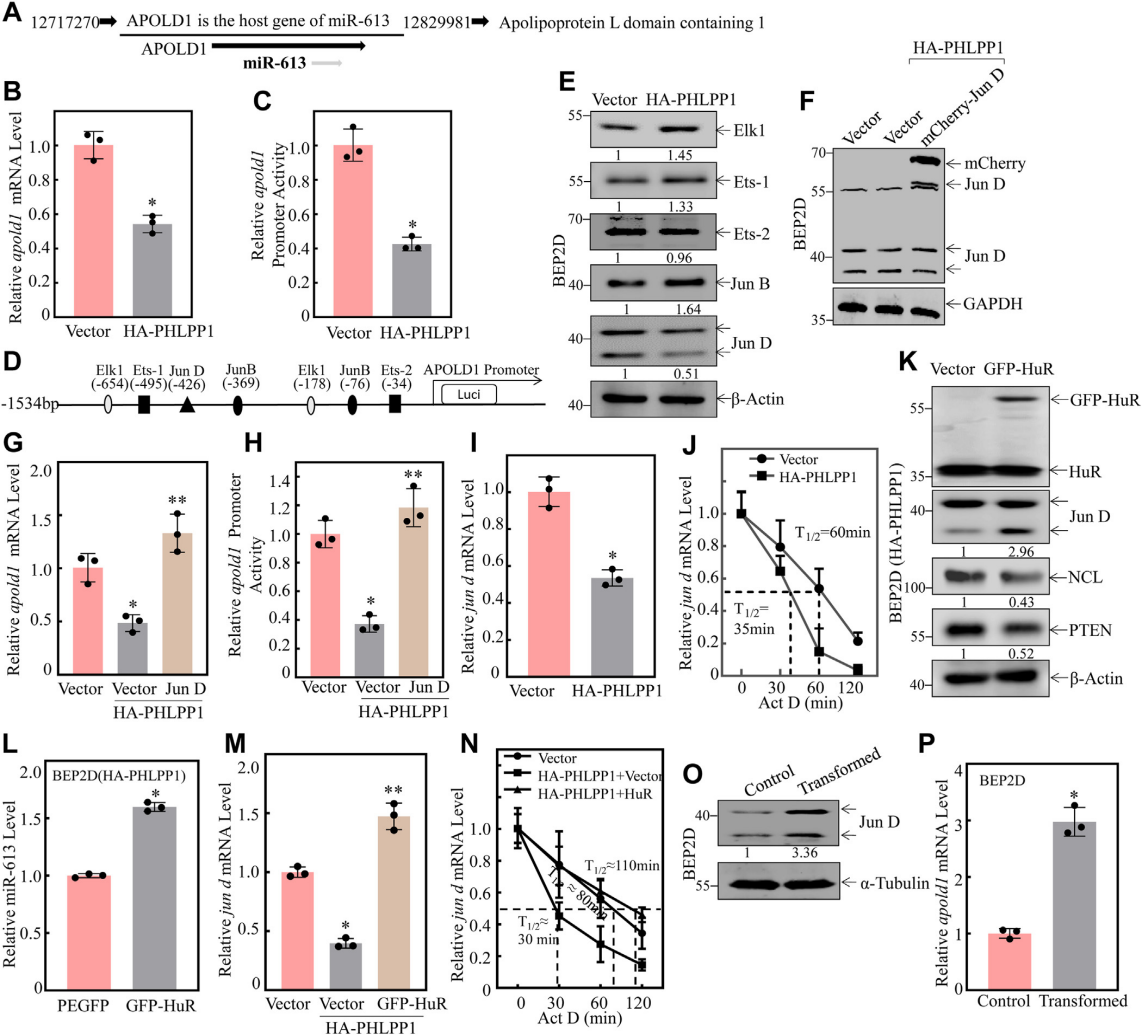
**Increase in Jun D transcriptionally regulates miR-613 expression during NNK-induced malignant transformed BEP2D cells.***A*, APOLD1 is the host gene of miR-613. *B*, relative *apold1* mRNA level was evaluated by real-time PCR in BEP2D(Vector) *vs.* BEP2D(HA-PHLPP1) cells. The *β-actin* was used as the mRNA loading control. *C*, the apold1 promoter-driven luciferase reporter constructs, together with TK, were stably transfected into BEP2D(Vector) *vs.* BEP2D(HA-PHLPP1), respectively. The transfected cells were extracted to evaluate the luciferase activity after 48 h, and the results are presented as relative apold1 promoter activity. The asterisk (∗) indicates a significant decrease of apold1 promoter transcriptional activity in BEP2D(HA-PHLPP1) compared to BEP2D(Vector) cells (*p* < 0.05). *D*, potential transcriptional factor binding sites in the APOLD1 promoter region (−1534∼+0) were analyzed using the TRANSFAC 8.3 engine online. *E*, Western blotting was used to detect Elk1, Ets-1, Ets-2, JunB, and JunD protein expression in the cells. β-Actin was used as a protein loading control. *F*, Western blotting was used to detect the overexpression efficiency of Jun D protein expression in BEP2D(HA-PHLPP1) cells. GAPDH was used as a protein loading control. *G*, relative *apod1* mRNA level was evaluated by real-time PCR in the indicated cells after Jun D overexpression. The asterisk (∗) indicates a significant decrease compared to BEP2D(Vector) cells (*p* < 0.05). The asterisk (∗∗) indicates a significant increase compared to BEP2D(HA-PHLPP1/Vector) cells (*p* < 0.001). The *β-actin* was used as the mRNA loading control. *H*, the apold1 promoter-driven luciferase reporter constructs, together with TK, were stably transfected into the indicated cells. The transfected cells were extracted to evaluate the luciferase activity after 48 h, and the results are presented as relative phlpp1 promoter activity. The asterisk (∗) indicates a significant decrease compared to BEP2D(Vector) cells (*p* < 0.05). The asterisk (∗∗) indicates a significant increase compared to BEP2D(HA-PHLPP1/Vector) cells (*p* < 0.001). *I*, relative *jun d* mRNA level was evaluated by real-time PCR in BEP2D(Control) *vs.* BEP2D(HA-PHLPP1) cells. The *β-actin* was used as the mRNA loading control. *J*, relative *jun d* mRNA stability was evaluated by real-time PCR in the indicated cell cells after treatment with Act D for indicated times. *K*, GFP-HUR or its control vector was transfected into BEP2D(HA-PHLPP1) cells, and the indicated stable transfectants were subjected to Western Blot to determine JunD, NCL, and PTEN protein expression as indicated. β-Actin was used as the protein loading control. *L*, qPCR was performed to assess the effect of overexpression GFP-HUR on miR-613 expression in BEP2D(HA-PHLPP1) cells. The asterisk (∗) indicates a significant change (*p* < 0.05). The *U6* was used as the miRNA loading control. *M*, relative *jun d* mRNA stability was evaluated by real-time PCR in the indicated cell cells. The asterisk (∗) indicates a significant inhibition compared to DEP2D(Vector) cells (*p* < 0.05); The asterisk (∗∗) indicates a significant increase compared to BEP2D(HA-PHLPP1/Vector) cells (*p* < 0.001). The *β-actin* was used as the mRNA loading control. *N*, relative *jun d* mRNA stability was evaluated by real-time PCR in the indicated cell cells after treatment with Act D for indicated times. *O*, Western blotting was used to detect Jun D protein expression in BEP2D transformed cells. α-Tubulin was used as a protein loading control. *P*, relative *apold1* mRNA level was evaluated by real-time PCR in BEP2D transformed cells. The *β-actin* was used as the mRNA loading control. All experiments were performed at least three replicates.

Intragenic miRNAs and their host genes share the same promoter. miR-613 is conserved and located within the intronic region of its host gene, APOLD1 (Fig. 5*A*), so we determined the effect of HA-PHLPP1 overexpression on *apold1* mRNA levels. The results showed that *apold1* mRNA expression was significantly suppressed in BEP2D(HA-PHLPP1) cells compared with that in BEP2D(Vector) cells (Fig. 5*B*). To evaluate the molecular mechanisms underlying this *apold1* mRNA downregulation, a luciferase reporter driven by the *apold1* promoter was employed. As shown in Figure 5*C*, overexpression of HA-PHLPP1 significantly impaired the transcriptional activity of the *apold1* promoter, indicating that PHLPP1 inhibits *apold1*/miR-613 expression at the transcriptional level. Therefore, the potential transcription factor binding sites in the APOLD1 promoter region were analyzed using the TRANSFAC 8.3 engine online program. The results showed that there were multiple potential transcription factor binding sites, including Jun D, Jun B, Elk1, Ets-1, and Ets-2, in the APOLD1 promoter region (Fig. 5*D*). To identify the transcription factors involved in *apold1*/miR-613 regulation, the protein expressions of Jun D, Jun B, Elk1, Ets-1, and Ets-2 were examined in BEP2D(HA-PHLPP1) cells and BEP2D(Vector) cells. As shown in Figure 5*E*, the level of Jun D, but not ElK-1, Ets-1, and Jun B, was suppressed in BEP2D(HA-PHLPP1) cells in comparison to that in BEP2D(Vector) cells, consistent with the changes in *apold1* mRNA and promoter activity in those transfectants. These results demonstrate that the downregulation of PHLPP1 promotes Jun D expression and may activate *apold1*/miR-613 transcription, thereby upregulating miR-613 expression in NNK-exposed BEP2D cells.

To determine the effect of Jun D downregulation on PHLPP1 inhibition of *apold1* mRNA and its promoter transcriptional activity, the mCherry-Jun D expression construct was stably transfected into BEP2D(HA-PHLPP1) cells (Fig. 5*F*). The results indicated that the inhibition of *apold1* mRNA (Fig. 5*G*) and promoter activity (Fig. 5*H*) by HA-PHLPP1 overexpression was significantly reversed by ectopic expression of mCherry-Jun D. The results strongly suggested that Jun D did act as a downstream effector of PHLPP1 responsible for its regulation of *apold1*/miR-613 transcription and expression. To elucidate the molecular mechanisms underlying PHLPP1 modulation of Jun D expression, the mRNA level of *jun d* was evaluated in BEP2D(HA-PHLPP1) vs. BEP2D(Vector) cells. As shown in Figure 5*I*, *jun d* mRNA levels were significantly reduced in HA-PHLPP1 overexpressed transfectants compared to those in scramble vector transfectants. To determine whether PHLPP1 regulated *jun d* mRNA stability, mRNA stability assays were performed in the presence of Act D to inhibit new mRNA synthesis in BEP2D (HA-PHLPP1) vs. BEP2D(Vector) cells. Upon inhibition of any new mRNA transcription due to the presence of Act D, *jun d* mRNA degradation rates in BEP2D(HA-PHLPP1) cells were significantly increased compared to those in BEP2D (Vector) cells (Fig. 5*J*). These results clearly indicated that PHLPP1 downregulated *jun d* mRNA stability in intact cells. RNA-binding protein (RBP) HuR has been reported to bind to *jun d* mRNA and regulate its mRNA stability (23). Since our results showed that PHLPP1 overexpression dramatically attenuated HuR protein expression (Fig. 3*E*), a GFP-HuR expression construct was stably transfected into BEP2D(HA-PHLPP1) cells. The results indicated that overexpression of HuR in BEP2D(HA-PHLPP1) cells significantly increased Jun D protein expression, and decreased NCL and PTEN expression (Fig. 5*K*). Moreover, HuR overexpression promoted miR-613 expression in BEP2D(HA-PHLPP1) cells (Fig. 5*L*), reversed PHLPP1 inhibition of *jun d* mRNA expression (Fig. 5*M*) and mRNA stability (Fig. 5*N*). Consistently, the upregulation of Jun D and *apodl1* expression were also observed in chronic NNK-transformed BEP2D cells *in vitro* (Fig. 5, *O* and *P*) Collectively, our results suggested that HuR directly upregulates *jun d* mRNA by increasing its stability.

### NNK decreased PHLPP1 transcription *via* PP2A/c-jun axis

**Figure 6:**
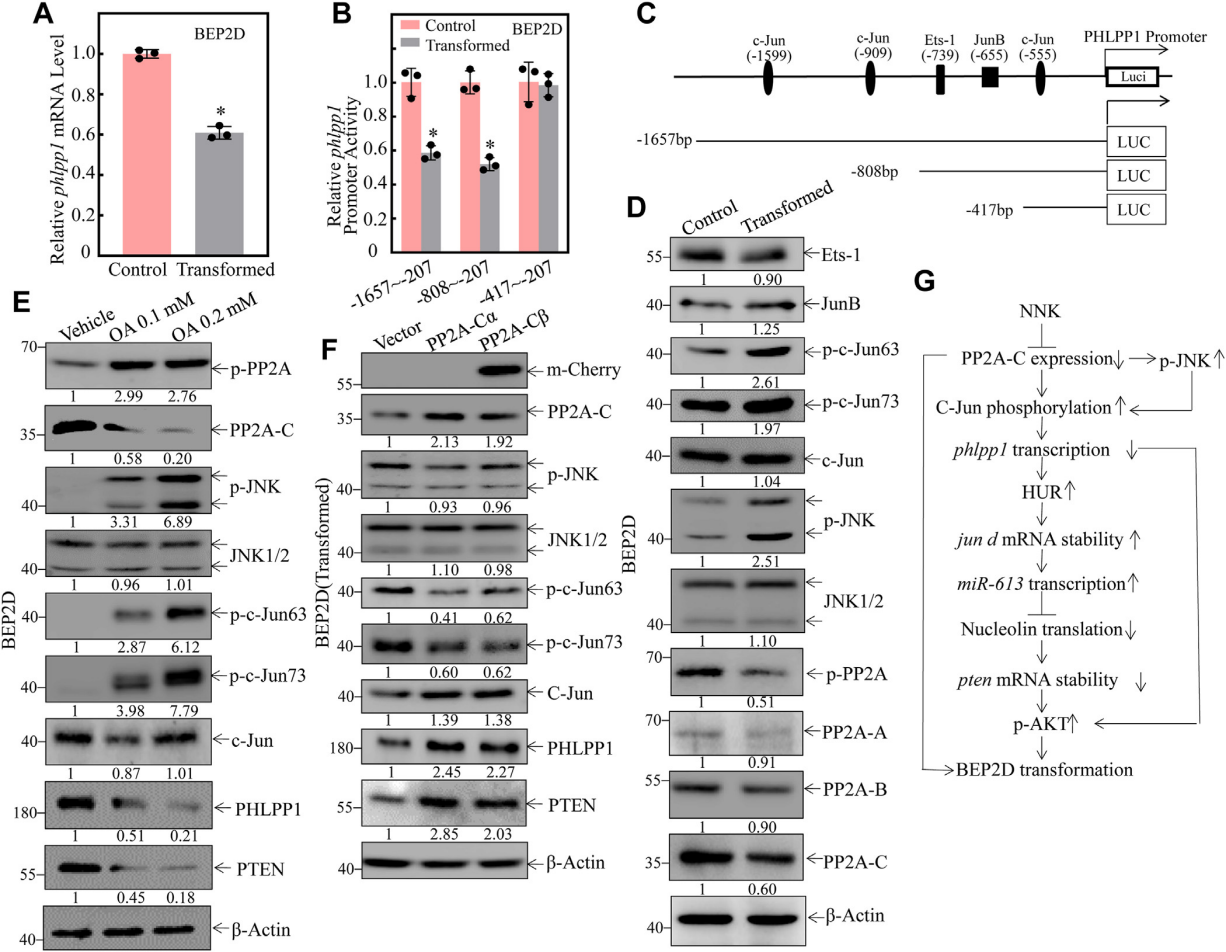
**NNK increased phosphorylation of c-Jun at Ser-63/Ser-73 through down-regulating PP2A-C and up-regulating phosphorylation of JNK.***A*, relative *phlpp1* mRNA level was evaluated by real-time PCR in BEP2D(Control) *vs.* BEP2D(transformed) cells. *β-actin* was used as the mRNA loading control. *B*, the various phlpp1 promoter-driven luciferase reporter constructs, together with TK, were stably transfected into BEP2D (Control) and BEP2D (transformed) cells, respectively. The transfected cells were extracted to evaluate the luciferase activity after 48 h, and the results are presented as relative *phlpp1* promoter activity. The asterisk (∗) indicates a significant decrease in comparison to untransformed control cells (*p* < 0.05). *C*, a schematic illustration of the construction of *phlpp1* promoter-driven luciferase reporter constructs. *D*, Western blotting was used to detect Ets-1, JunB, phosphorylated c-Jun at Ser63, phosphorylated c-Jun at Ser73, phosphorylated JNK, JNK, phosphorylated PP2A, PP2A-A, PP2A-B, PP2A-C subunit and c-Jun protein expression in the indicated cells. β-Actin was used as a protein loading control. *E*, BEP2D cells (2 × 10^5^) were seeded into each well of 6-well plates. After synchronization, cells were treated with the indicated concentration of okadaic acid (OA) for 8 h in a traditional medium and then extracted for Western blotting to determine the protein levels of phosphorylated PP2A, PP2A-C subunit phosphorylated JNK, JNK1/2, phosphorylated c-Jun at Ser63, phosphorylated c-Jun at Ser73 and c-jun. β-Actin was used as a protein loading control. *F*, overexpression of PP2A-Cα and PP2A-Cβ in BEP2D(transformed) cells and Western blotting were used to detect phosphorylated PP2A, PP2A-C subunit phosphorylated JNK, JNK1/2, phosphorylated c-Jun at Ser63, phosphorylated c-Jun at Ser73 and c-jun protein expression in the cells. β-Actin was used as the protein loading control. *G*, the proposed mechanisms underlying the downregulation of PHLPP1 contribute to the NNK-induced malignant transformation of human bronchial epithelial cells through regulating *pten* mRNA stability. All experiments were performed at least three replicates.

To reveal the molecular mechanisms for the decreased PHLPP1 after the NNK exposure, we investigated the *phlpp1* mRNA levels in BEP2D(control) and BEP2D(transformed) cells. As shown in Figure 6*A*, the level of *phlpp1* mRNA in BEP2D(transformed) cells was lower compared to BEP2D(control) cells. To test the possibility of transcriptional regulation, the *phlpp1* promoter-driven luciferase reporters (containing incrementally deleted sequences in the −1657/−207 region), were stably transfected into BEP2D(transformed) and BEP2D(Vector) cells (Fig. 6*B*). The results showed that the *phlpp1* promoter transcriptional activity was significantly impaired in the transfectant with the −1657/−207 and −808/−207 luciferase reporter, while there is no difference in −417/−207 luciferase reporter, suggesting that the transcription factor binding sites at −808/−207 domain are crucial for *phlpp1* transcription. Moreover, the potential transcription factor binding sites was predicted by bioinformatics software in the *phlpp1* promoter region at −808/−207 (Fig. 6*C*). As shown in Figure 6*D*, phosphorylation of C-Jun at Ser-63/Ser-73 was activated during NNK-induced malignant transformation of BEP2D cells compared to BEP2D(control) cells. However, the transcription factors Jun B and Ets-1 did not show a significant difference. According to our previous study, the *phlpp1* promoter (−808/−417 domain) contains putative C-Jun binding sites, and activation of C-Jun phosphorylation represses *phlpp1* transcription in Beas2b cells (24). Therefore, we anticipated that C-Jun might be the transcription factor that negatively regulates *phlpp1* promoter transactivation through its direct binding.

Phosphorylation of Ser-63 and Ser-73 in the NH2-terminal transactivation domain of c-Jun, primarily mediated by c-Jun NH2-terminal kinases (JNKs), plays a critical role in enhancing the transcriptional activity of c-Jun. Additionally, our previous study identified PP2A as a regulator of C-Jun phosphorylation at Ser-63/Ser-73 (25). Therefore, we compared the expression of JNK and PP2A (PP2A-A, PP2A-B, and PP2A-C) in BEP2D(transformed) cells relative to BEP2D(control) cells. As shown in Figure 6*D*, PP2A-C expression and P-PP2A were significantly decreased in BEP2D(transformed) cells compared to BEP2D(control) cells, while there were no significant differences in PP2A-A and PP2A-B expression. P-JNK, but not total JNK expression, was increased in NNK-transformed BEP2D cells in comparison to that observed in BEP2D(control) cells (Fig. 6*D*). Next, to investigate whether inhibition of PP2A-C activity could lead to an increase in C-Jun phosphorylation at Ser63/73 expression, we treated BEP2D cells with the PP2A inhibitor okadaic acid (OA). The results showed that inhibition of PP2A activity by OA treatment led to a marked induction of C-Jun phosphorylation at Ser63/73 and inhibition of PHLPP1 and PTEN in BEP2D cells (Fig. 6*E*). Furthermore, we constructed overexpression constructs for PP2A-Cα and PP2A-Cβ in BEP2D(transformed) cells to investigate their influence on C-Jun phosphorylation at Ser63/73 expression. The results showed that the overexpression of PP2A-Cα and PP2A-Cβ inhibited NNK-induced JNK phosphorylation/activation and C-Jun phosphorylation at Ser63/73 (Fig. 6*F*). Additionally, the overexpression of PP2A-Cα and PP2A-Cβ reversed NNK-induced downregulation of PHLPP1 and PTEN expression (Fig. 6*F*). Collectively, our results demonstrate that inactivation of PP2A-C is crucial for C-Jun phosphorylation at Ser63/73 in NNK-induced transformation cells. Taken together, our results suggest that NNK exposure increases c-Jun phosphorylation at Ser-63/Ser-73 through downregulation of PP2A-C activity and upregulation of JNK phosphorylation, leading to decreased PHLPP1 transcription and in turn promotes PTEN expression (Fig. 6*G*).

## Discussion

Despite the well-documented adverse effects of NNK in lung cancer, the molecular mechanisms involved in NNK-mediated pathogenesis remain elusive. In this study, we discovered the role of PHLPP1 and PTEN, two tumor suppressors, in the NNK-induced malignant transformation of human bronchial epithelial cells (BEP2D). Additionally, we delineated the regulatory relationships between PHLPP1 and PTEN in NNK-induced cell transformation. Based on the data generated in our study, Figure 6*G* depicts a proposed model of NNK-induced malignant transformation of human bronchial epithelial cells (BEP2D) through regulation of the PP2AC/PHLPP1/HUR/Jun D/miR-613/NCL/PTEN axis.

PTEN, a tumor suppressor that is widely expressed throughout the body tissues (26), catalyzes the dephosphorylation of the third phosphate of the inositol ring in PIP3, resulting in the inhibition of Akt (27, 28). Previous studies have shown that cigarette smoke extract (CSE) suppressed PTEN levels through miR-21 overexpression (29). However, the role of PTEN under NNK exposure has never been explored. Our current studies demonstrate that PTEN plays a crucial role in NNK-induced malignant transformation of human bronchial epithelial cells. We showed that NNK downregulated PTEN protein expression and overexpression of PTEN reversed the NNK-induced malignant transformation of human bronchial epithelial cells. These findings support the association between NNK exposure and lung cancer development in cigarette smokers.

PHLPP, a tumor suppressor, blocks growth factor-induced signaling in cancer cells. PHLPP1 and PHLPP2 are the two main isoforms of PHLPP, which are important regulators of Akt serine-threonine kinases and protein kinase C (PKC) isoforms (18, 30). Our studies indicate that PHLPP1, but not PHLPP2, plays a significant role in the NNK-induced malignant transformation of human bronchial epithelial cells. Additionally, we found that the downregulation of PHLPP1 increases Akt activation due to NNK exposure. Our results are consistent with the previous study that nicotine and the tobacco-specific carcinogen NNK activated the Akt serine/threonine kinase in human airway epithelial cells (31). Therefore, our results further indicate that Akt is known to be a tumor promoter, suggesting that the negative regulator PHLPP1 may act as a tumor suppressor. Our further mechanistic elucidation indicates that PHLPP1 affects PTEN expression after NNK exposure, but not PTEN-regulated PHLPP1 expression. While the downregulation of PHLPP1 and PTEN in lung cancer tissues has been reported previously (32, 33), the current study adds novelty by: 1), Demonstrating the direct effects of NNK exposure on PHLPP1 and PTEN expression. Uncovering a new molecular pathway involving the regulation of PTEN by PHLPP1 and the HUR/Jun D/miR-613/NCL axis, which has not been widely explored in the context of tobacco-related lung carcinogenesis; 2), Prior studies have explored the individual roles of PHLPP1 and PTEN in cancer, including their downregulation in response to carcinogenic factors. However, the specific interaction between these two tumor suppressors in the setting of NNK exposure, along with the detailed mechanistic pathways identified here, adds a new layer of understanding that was lacking in previous research.

In general, the phosphorylation of serine 63 and 73 increased the transcriptional activity of c-Jun (34). In this study, NNK downregulates *phlpp1* transcription by increasing the c-Jun phosphorylation at 63 and 73. Besides, our previous study also discovered that c-Jun negatively regulates *phlpp1* transcription (24). Similarly, the study has been reported that activation of the transcription factor c-Jun negatively regulates mdr-1 gene expression in salvicine-treated MDR K562/A02 cells (35). So, these results indicate that c-Jun can act as a negative regulatory factor for *phlpp1* promoter transcription. There are several reasons for this discrepancy. AP-1 is a complex composed of Jun and Fos family members. First and foremost, multiple proteins can form complexes that bind to AP-1 sites. When Jun interacts with numerous other transcription factors, this transcriptional crosstalk modulates the activities of Jun and its partners (36, 37). Second, induction of c-jun expression does not always mean activation of AP-1 (38). Therefore, these observations highlight the complex versatility of c-Jun, prompting us to determine the various roles of c-Jun in transcription regulation in future studies.

This study reveals the mechanisms of NNK treatment on lung tumorigenesis. Although these results are highly relevant, it is important to note that the way NNK undergoes metabolic processes *in vivo* and *in vitro* has not been rigorously studied. In the future, we plan to continue to investigate the NNK metabolic mechanisms through the cytochrome P450 pathway.

**Figure 7:**
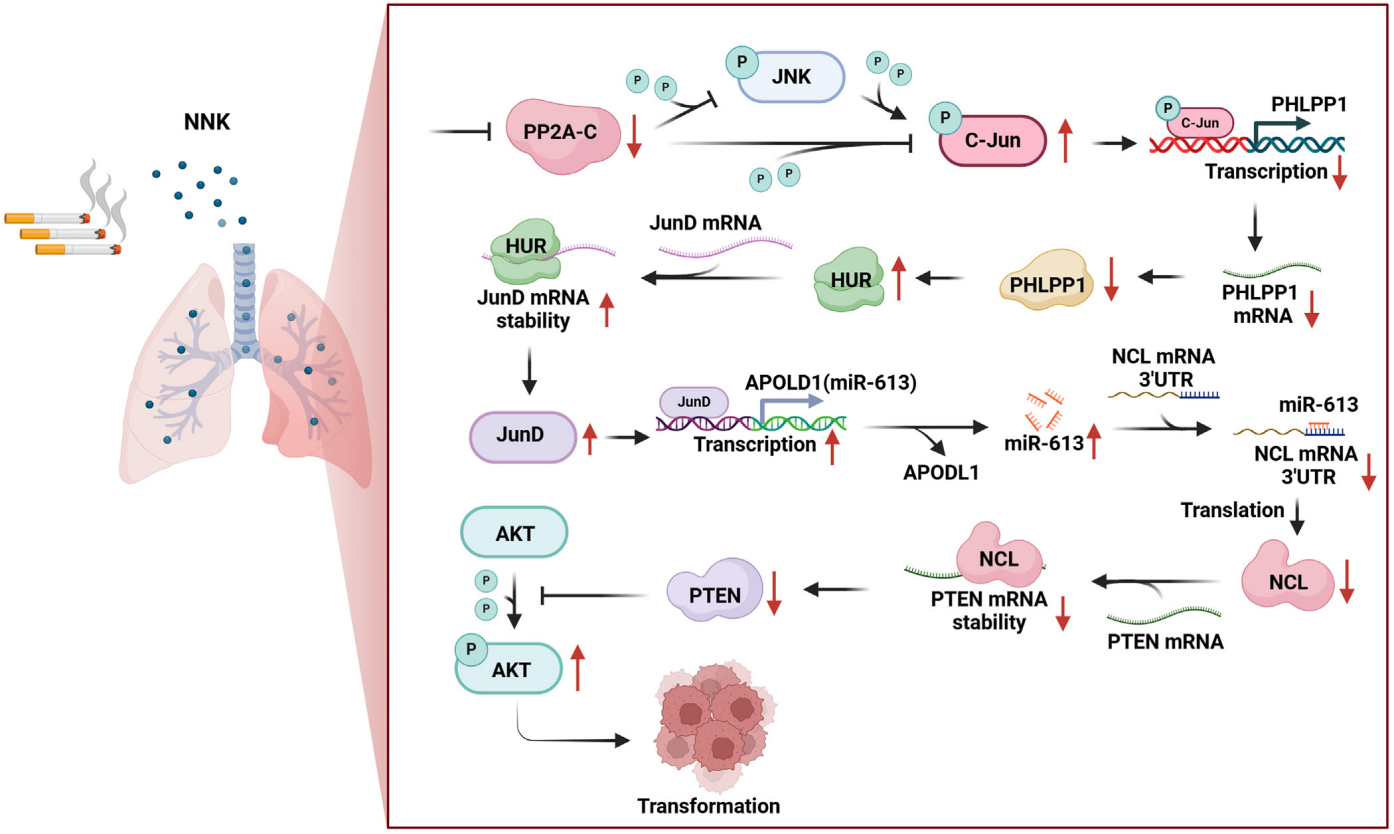
**The schematic diagram of NNK-induced malignant transformation of human bronchial epithelial cells.** Briefly, NNK inhibited PP2A-C activity, which directly attenuated c-Jun phosphorylation at Ser63/73, and subsequently inhibited the PHLPP1 transcription and expression. The downregulation of PHLPP1 reduced *pten* mRNA stability and expression through the HUR/Jun D/miR-613/NCL axis, resulting in malignant transformation of human bronchial epithelial cells.

In conclusion, our results indicate how exposure to NNK, a tobacco-specific carcinogen, affects the expression of two key tumor suppressors - PHLPP1 and PTEN - during lung carcinogenesis. The study finds that NNK exposure results in the downregulation of both PHLPP1 and PTEN in human bronchial epithelial cells and mouse lung tissues. We further demonstrate that overexpressing PHLPP1 or PTEN can alleviate NNK-induced malignant transformation in cells. Mechanistic studies reveal that NNK inhibits PP2A-C activity, affecting c-Jun phosphorylation and subsequently reducing PHLPP1 transcription, which then decreases PTEN mRNA stability through a novel molecular axis (Fig. 7). This study makes several contributions to the field of lung cancer research, specifically in the context of tobacco-induced carcinogenesis: (1) novel Discovery of the PHLPP1-PTEN Interaction during NNK-induced lung carcinogenesis, which provides new insight into the molecular events that occur early in lung cancer development following tobacco exposure; (2) mechanistic Insights into Tobacco Carcinogenesis: The research provides a novel mechanistic understanding of how NNK affects tumor suppressors, including the role of PP2A-C and c-Jun phosphorylation. This pathway has not been fully explored in the context of NNK exposure and cancer, highlighting potential targets for therapeutic intervention in lung cancer caused by smoking; (3) potential Clinical Relevance: Since both PHLPP1 and PTEN are well-documented tumor suppressors that are frequently downregulated in lung cancer, understanding the mechanisms behind their dysregulation could have significant clinical implications, including new diagnostic or therapeutic approaches for early-stage lung cancer in smokers.

## Experimental procedures

### Plasmids, reagents, and antibodies

The HA-PHLPP1 plasmid was obtained from Addgene. The shNCL plasmid was purchased from OpenBiosystem. The EGFP-PTEN fusion protein expression plasmid was donated by Dr Xia Zhang (Neuropsychiatry Research Unit). NNK was purchased from Toronto Research Chemicals. The transfection reagent was purchased from SignaGen Laboratories. The antibodies specific against p-Akt Ser473, p-Akt Thr308, Akt, PTEN, PP2A-A, PP2A-B, PP2A-C subunit, and c-Jun were purchased from Cell Signaling. The antibodies against phospho-PP2A Y307 were bought from Epitomics. The PHLPP1 and PHLPP2 antibodies were purchased from the Bethyl Laboratories.

### Cell culture and the plasmid transfection

The BEP2D cells were cultured in serum-free LHC-8 medium (Biosource International, Camarillo, CA), which contains epidermal growth factors. The *mycoplasma* test for BEP2D cells was negative. BEF2D cells were treated with 0.2 μg/ml or 0.4 μg/ml. Plasmid transfection was performed according to the instructions of the PolyJetTM DNA *in vitro* transfection kit. 24 h after transfection, the transfected cell, including BEP2D(HA-PHLPP1), BEP2D(PTEN-GFP), BEP2D(shNCL), BEP2D(miR-219), BEP2D(miR-613), BEP2D(PP2A-Cα), BEP2D(PP2A-Cβ), were treated with the antibiotic puromycin(0.3 μg/ml) (Alexis, BML-A260–0050) or G418 (500 μg/ml; Invitrogen, 10131027) for stable selection to obtain positive transfected cells.

### Animal experiments

Lung tissue from animals exposed to cigarette extract was donated by Dr Xinxin Ding (Wadsworth Center, New York State Department of Health, Albany, New York). The exposure protocol used to induce the development of lung inflammation performed as described in the studies previously (39). Mice were exposed to either HEPA-filtered air (FA) or environmental tobacco smoke ETS for 2 weeks. And then the animals were euthanized using carbon dioxide 24 h after completion of the last FA or ETS exposure. All studies involving animals are approved by the Institutional Animal Welfare and Care Committee of Wenzhou Medical University.

### Western blot analysis

Cell extracts were subjected to Western blotting using specified antibodies in strict adherence to previously established protocols (40). Visualization and capture of the resultant immuno-reactive bands were facilitated through scanning *via* a Typhoon FLA 7000 imager. The protein bands in Western blot images were quantified using the Image J software.

### Luciferase assay

Luciferase reporter assays used in this study were performed as previously described (41). PHLPP1, APOLD1, or NCL promoter luciferase reporter constructs, were transiently transfected into the BEP2D cells, in combination with the pRL-TK vector (Promega) as an internal control.

### Quantitative RT-PCR for mRNA assay

Total RNA was isolated utilizing TRIzol reagent (Invitrogen) and reverse transcribed into cDNA. For RNA stability, in the presence of actinomycin D (RNA synthesis inhibitor, 10 mM), mRNA degradation rates in BEP2D cells and transfectants were determined for the indicated time periods. Specific primer pairs were deployed for the amplification of targeted genes as delineated in the corresponding figures and as previously described (42). The primers used in this study are listed in Table S1.

### Quantitative real-time PCR for miRNA

Total miRNA extraction from cells was performed according to the instructions of the miRNeasy Mini Kit (Qiagen). From this, aliquots of total miRNA (2 μg) were used for reverse transcription. 7900HT Fast Real-time PCR system (Applied Biosystems) was used to analyze the miR-1, miR-140, miR-194, miR-203, miR-206, miR-219, and miR-613 expressions. The relative expression levels of each miRNA were calculated according to 2^−ΔΔCT^. Sequence alignments of NCL 3′-UTR with seed regions of putative microRNAs were listed in Table S2. U6 was used as a control.

### RNA immunoprecipitation (RNA-IP) assay

The RNA-IP assay was performed as described in the studies previously (43). RT-PCR assay was conducted to detect the mRNA expression presented in the immune-complex.

### Anchorage-independent growth assay in soft agar

NNK at 0.4 μg/ml final concentration was used to treat BEP2D cells for 4 weeks to induce cell transformation. Anchorage-independent growth assay in soft agar (soft agar assay) was performed, and the specific experimental procedure is referred to in our previous study (44). Colonies were then identified using a CKX41 light microscope (Olympus). The results are expressed as the mean ± standard deviation (SD) of three independent experiments.

### CCK-8 assay

Cell viability was measured using the Cell Counting Kit-8(Sigma-Aldrich, Shanghai, China) according to the manufacturer’s instructions. Briefly, BEP2D cells were seeded in 96-well plates at a density of 8000 cells per well. Different concentrations of NNK were added and incubated at 37 °C for 48 h. Then, CCK-8 was added and incubated for 3 h. The optical density was measured at 450 nm. Experiments were performed at least three biological replicates.

### Statistical methods

The student's *t* test was used to determine the significance of differences between various experimental groups. The results are expressed as mean ± SD. All experiments were performed with at least three biological replicates. The differences were significant at *p* < 0.05.

## Data availability

Data will be provided after reasonable request.

## Supporting information

This article contains supporting Information.

## Conflict of interest

The authors declare that they have no conflicts of interest with the contents of this article.
