# Supplementary material for: Downregulation of the phosphatase PHLPP1 contributes to NNK-induced malignant transformation of human bronchial epithelial cells (HBECs)
Source: J Biol Chem. 2025 Jan 23;301(3):108221. doi: 10.1016/j.jbc.2025.108221 (PMC11889559; doi:10.1016/j.jbc.2025.108221)
Supplement: Supporting information [file mmc1.docx]

Supplementary Table S1 Primer used in the article.

| Gene | Forward 5’-3’ | Reverse 5’-3’ |
| --- | --- | --- |
| PTEN | ACA CCG CCA AAT TTA ACT GC | TAC ACC AGT CCG TCC CTT TC |
| PHLPP1 | ACACCGTGATTGCTCACTCC | TTCCAGTCAGGTCTAGCTCC |
| NCL | CCAGAAGCCAGCCATCCAA | ACCCAGTTTCCCGGGTCAGTA |
| JunD | AGTCCTCAGCCACGTCAACA | TCCAGCTTGTCGAGTCCTGG |
| APOLD1 | AACGCCTCCATCGCCCTGTA | CCTGGCTAACCTTGGTGTCCC |
| β-actin | ACAGAGCCTCGCCTTTGCC | GATATCATCATCCATGGTGAGCTGG |

Supplementary Table S2 Sequence alignments of NCL 3’-UTR with seed regions of putative microRNAs.

| NCL-3’UTR: 5’… (76) CAGAGCCUUCUGAGGACAUUCCA (98) …  has-miR-1: 3’ UAUGUAUGAAGAAAUGUAAGGU |
| --- |
| NCL-3’UTR: 5’… (97) AAGACAGUAUACAGUCCUGUGGU (119) …  hsa-miR-140: 3’ CAGGCACCAAGAUGGGACACCA |
| NCL-3’UTR: 5’… (42) ACUCUGGGGUUUUUACUGUUACC (64) …    has-miR-194: 3’ AGGUGUACCUCAACGACAAUGU |
| NCL-3’UTR: 5’… (129) AAAUCCGUCUAGUUAACAUUUCA (151) …  has-miR-203: 3’ GAUCACCAGGAUUUGUAAAGU |
| NCL-3’UTR: 5’… (76) CAGAGCCUUCUGAGGACAUUCCA (98) …  has-miR-206: 3’ GGUGUGUGAAGGAAUGUAAGGU |
| NCL-3’UTR: 5’… (412) AGGACAAAUUAAAAGUCAACUCU (434) …  has-miR-219: 3’ GCCCUGCAGGUCUGAGUUGAGA |
| NCL-3’UTR: 5’… (76) CAGAGCCUUCUGAGGACAUUCCA (98) …  has-miR-613: 3’ CCGUUUCUUCCUUGUAAGGA |
